# A Medical Defense of Tobacco

**Published:** 1908-06

**Authors:** 


### A Medical Defense of Tobacco.

An attack upon what is termed the "popular prejudice" against the cigarette and an attempt to discourage all anti-tobacco legislation, appears in the editorial columns of the *American Medicine* (Philadelphia, March). Says the writer:

"The harmlessness of tobacco in moderation seems to be conceded by the great majority of physicians, and it is difficult to account for the numerous statements to the contrary, particularly in the school physiologies. The popular prejudice against the cigarette is still more amazing in view of the absence of any proof that it is harmful except to very immature boys. Of course, excessive use of tobacco has well-defined symptoms, and there are a few people to whom a very limited indulgence is excess, but in the hundreds of millions who use it in one form or another, there is astonishingly little evidence of injury.

"The usefulness of tobacco has received so little scientific attention that practically nothing is popularly known of this side of the question. Such a world-wide custom must serve some useful purpose, as it can be taken for granted that useless or harmful habits do not survive in any species of animal. It is our duty to find out what the benefit really is. The acute poisoning in those unaccustomed to it is so well described in the text-books as to need no comment; what is needed is knowledge of the effect of small amounts in those accustomed to it. The after-dinner cigar has been said to increase the flow of gastric secretion and hasten digestion, and yet that alleged fact has been vehemently denied and the reverse asserted. The real use of tobacco is in some obscure sedative effect upon the nervous system, particularly the higher cerebral cells, though the effect of larger indulgence is exciting to the point of delirium.

"Mankind has instinctively found that it is comforting in some way which no one can describe, and womankind—much to the astonishment of the Northern races—is discovering the same fact."

Deprivation of tobacco, the writer goes on to say, is the severest punishment for a convict, and an army without it may become inefficient to the point of demoralization and defeat. Very likely the work of civilians is also dependent upon its effect, so that if the trade were destroyed civilization would suffer. The author admits that these will seem rather radical views to non-users of tobacco, but its enormous consumption, he asserts, permits no other opinion. He goes on:

"Indeed, there is now and then a hint that the abstainers as a class do not sustain as heavy burdens as they could, nor as long as they could, if they had the assistance of tobacco. In the absence of exact data, these discussions are bootless, but such knowledge as we possess places the burden of proof upon those who claim that the drug is always harmful and never of use. The opposition to tobacco is wholly unaccountable—even the ethical statement that it is the first step to the saloon and brothel is disproved by those who have not taken those steps. The outcry against tea and coffee is readily understood, for most of it comes from those who have an alleged substitute to sell for these essentials of modern life.

"The effect of tobacco upon nearly grown boys has always been assumed to be harmful, and the majority of physicians would doubtless advise abstinence until full maturity. It is rather startling, then, to learn that Dr. George L. Meylan, physical director of Columbia University, has found that the students who use tobacco are taller, heavier, and stronger than the abstainers, and that the difference is more than would be accounted for by the slightly greater age of the former. It may not prove that the drug has been beneficial, but it does prove that we were wrong in assuming that boys were stunted by its use. The investigations of recent years have shattered a great many medical opinions of great age, and this seems to be another instance. What a comment it all is upon our very human propensity to form theories before we hear the facts. Hereafter let all theorists present their facts first and let the anti-cigarette laws wait until it is proved they are needed. At present they create ridicule and contempt of law, for they can not be enforced."—*Literary Digest.*

